# Eye-Movement-Controlled Wheelchair Based on Flexible Hydrogel Biosensor and WT-SVM

**DOI:** 10.3390/bios11060198

**Published:** 2021-06-16

**Authors:** Xiaoming Wang, Yineng Xiao, Fangming Deng, Yugen Chen, Hailiang Zhang

**Affiliations:** School of Electrical and Automation Engineering, East China Jiaotong University, Nanchang 330013, China; 2501@ecjtu.edu.cn (X.W.); 2019028080800015@ecjtu.edu.cn (Y.X.); c15070859515@163.com (Y.C.); 1860@ecjtu.edu.cn (H.Z.)

**Keywords:** flexible hydrogel biosensor, human-wheelchair interaction, eye movement detection, signal classification algorithm

## Abstract

To assist patients with restricted mobility to control wheelchair freely, this paper presents an eye-movement-controlled wheelchair prototype based on a flexible hydrogel biosensor and Wavelet Transform-Support Vector Machine (WT-SVM) algorithm. Considering the poor deformability and biocompatibility of rigid metal electrodes, we propose a flexible hydrogel biosensor made of conductive HPC/PVA (Hydroxypropyl cellulose/Polyvinyl alcohol) hydrogel and flexible PDMS (Polydimethylsiloxane) substrate. The proposed biosensor is affixed to the wheelchair user’s forehead to collect electrooculogram (EOG) and strain signals, which are the basis to recognize eye movements. The low Young’s modulus (286 KPa) and exceptional breathability (18 g m^−2^ h^−1^ of water vapor transmission rate) of the biosensor ensures a conformal and unobtrusive adhesion between it and the epidermis. To improve the recognition accuracy of eye movements (straight, upward, downward, left, and right), the WT-SVM algorithm is introduced to classify EOG and strain signals according to different features (amplitude, duration, interval). The average recognition accuracy reaches 96.3%, thus the wheelchair can be manipulated precisely.

## 1. Introduction

Data from the World Health Organization (WHO) indicate that wheelchairs are indispensable for 75 million people on a daily basis, which accounts for 1% of the world’s total population [1]. Those who are highly handicapped (e.g., ALS patients) are incapable of maneuvering their wheelchairs manually. Given this, it is significant to develop a novel method to assist them to control the wheelchair.

The hand gesture control [2,3,4,5] and voice control [6,7,8] has been explored by several scholars. However, the main challenge for hand gesture control is the laborious operation and misrecognition of gestures [9]. In addition, it is not applicable for people with limited limb movement. As for voice control, the speech commands are susceptible to ambient noise, which greatly reduces the practicality in noisy environments [10]. Multiple studies have demonstrated the utilization of video-based eye-tracking systems [11,12,13]. These systems require specific illumination conditions and are expensive, so it is not viable for large-scale applications [14]. Brain–computer interface (BCI) technology has been employed in human–wheelchair interactions previously [15,16,17,18]. The acquisition of Electroencephalogram (EEG) signals is usually realized by invasive electrodes, which brings great difficulty to implementation and may impact the physical health of the user [19].

Eye movement is a conscious and subjective behavior, and its directions can be identified by the analysis of electrooculogram (EOG). There are also some studies about eye movement control in wheelchairs [20,21,22,23,24,25,26]. EOG is a kind of electrophysiological method, which records the potential difference between the cornea and retina [27,28,29]. A wireless human–wheelchair interface has been exhibited in [20], where the EOG signal is measured by a soft electrode. This electrode is prepared by depositing Au on a membrane and transferring it into a specific pattern. Huang et al. placed three metal electrodes on the left eyebrow, left mastoid, and right mastoid, respectively, to record vertical EOG signals [22]. In the above studies, the electrodes are rigid and have poor biocompatibility, which may cause allergies or irritations during direct contact with skin.

Signal classification is the basis for identifying the diverse movement states of eyeballs. Various algorithms have emerged in the past decade, including the Hidden Markov Model (HMM) [30,31,32,33], transfer learning [34,35,36,37], and linear classifiers [38,39,40,41]. A hierarchical HMM statistical algorithm [33] was utilized to classify ternary eye movements and the classification result of fixations, saccades and smooth pursuits was evaluated as “good”. However, this model cannot label multiple features simultaneously, which limits the efficiency of classification. Abdollahpour et al. reported a transfer learning convolutional neural network (TLCNN) and applied it in the classification of the EEG signal’s feature set [34]. It trains a general classifier instead of an optimal one, so the accuracy of the classification is relatively low.

Linear discriminant analysis (LDA) and threefold support vector machine (SVM) approaches were used to classify six natural facial expressions and the designed framework minimized the generation of false labels and increased the classification accuracy [38]. Nevertheless, the traditional SVM has problems of complex calculation and long running times when dealing with the superimposed signals with linear inseparability [42,43,44]. Therefore, it cannot be implemented in the real-time classification of electrophysiological signals independently.

In this paper, an eye-movement-controlled wheelchair prototype based on flexible hydrogel biosensor is presented. The proposed biosensor owns high deformability and excellent biocompatibility. Moreover, Kalman filtering and Wavelet Transform-Support Vector Machine (WT-SVM) algorithms are applied in the processing and classification of EOG and strain signals to distinguish various eye movement states. The algorithm’s recognition rate of eye movement reaches 96.3%, which ensures the high-precision control of the wheelchair.

## 2. Design and Method

### 2.1. Human–Wheelchair Interaction

To achieve eye movement control in wheelchairs, we have performed the following three aspects of work: the fabrication of a flexible hydrogel biosensor, signal classification, and the manipulation of a wheelchair. The biosensor is responsible for collecting the wheelchair user’s EOG and strain signals. After being processed by the peripheral circuit, signals will be input into the laptop (Surface Pro 7) in digital form. The application of the classification algorithm enables different eye movement states to be identified. Eventually, the laptop generates instructions to drive stepper motors, and then control the wheelchair.

**Figure 1:**
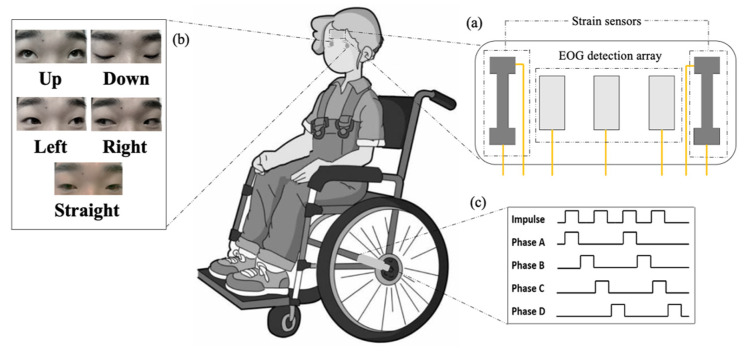
Eye-movement-controlled wheelchair prototype based on a flexible biosensor. (**a**) Structure of flexible biosensor. (**b**) Diverse movement states of eyeballs. (**c**) Pulse signal for driving stepper motors.

Five eye movement states (up, down, left, right, and straight) correspond to the different mobile modes of the wheelchair: ‘up’ to move forward, ‘down’ to move back, ‘left’ to turn left, ‘right’ to turn right, and ‘straight’ to stay still. The overall framework of the study is presented in Figure 1.

### 2.2. Fabrication of A Flexible Biosensor

The flexible biosensor is comprised of three layers. The HPC/PVA (Hydroxypropyl cellulose/Polyvinyl alcohol) layer is sandwiched between two PDMS (Polydimethylsiloxane) layers. Due to its dielectric and biocompatible properties, the PDMS substrate is in direct contact with epidermis to insulate electrical interference [45,46]. As a sensing layer, the function of the HPC/PVA hydrogel membrane is to collect the electrophysiological signals.

The fabrication procedures of the PDMS substrate are as follows: mix PDMS aqueous dispersion (Shanghai Macklin Biochemical Technology Co., Ltd., Shanghai, China) with a coagulant at the ratio of 10:1 in the flask and stir evenly; set it aside until all the bubbles disappear; coat the mixture onto a glass slide; transfer the glass slide on a heating plate (IKA, C-MAG HP 4); and heat it at 75 °C. About half an hour later, a piece of PDMS film can be detached from the glass slide.

The conductive hydrogel membrane can be manufactured by the steps below. Add 5 mL DMSO (Dimethyl sulfoxide, Aladdin Co., Shanghai, China) and 0.5 g HPC (Aladdin Co., Shanghai, China) to 20 mL DI water and heat the mixture in a water bath (70 °C) with constant stirring. After 15 min, add 3 g PVA (Sigma-Aldrich Co., Saint Louis, MO, USA) to the mixed solution. Adjust the temperature to 85 °C and continue heating for two hours. Pour the mixed solution into a metal groove and let it cool down naturally. Transfer the cooled solution to the refrigerator at −20 °C and take it out after half an hour. When rising to room temperature, put it into the refrigerator again. Through three cycles, the HPC/PVA hydrogel membrane can be peeled off from the bottom of the metal groove.

The smaller size of biosensor guarantees an unobtrusive experience, but it has limited accuracy and sensitivity. To obtain a preferred dimension of the PDMS substrate and hydrogel membrane, we designed an elastic cantilever device according to Equation (1):

(1) $$K=\frac{EWT^{3}}{6L^{3}}$$

where *K* is the elastic constant of the cantilever, *E* is the tensile modulus, and *W*, *T*, and *L* represent the width, thickness, and length of the fabricated film, respectively.

Cut the hydrogel membrane (0.2 mm in thickness) into rectangular (10 × 10 mm^2^) and I-shaped (10 × 20 mm^2^) shapes respectively. Then, attach the I-shaped membrane to the left and right sides of the fabricated PDMS substrate (60 × 25 mm^2^, 0.3 mm in thickness), and affix three pieces of rectangular membranes equally spaced to the middle of it. Finally, encapsulate the PDMS substrate and the HPC/PVA membrane with epoxy resin to enhance the adhesion [47].

In order to determine whether the designed biosensor with the preferred dimensions can adapt to the deformation of epidermis, we studied its load-deflection characteristic (Equation (2)).

(2) $$P=\frac{\pi^{4}ET^{3}}{6\left(1-\nu^{2}\right)L^{4}}*c$$

**Figure 2:**
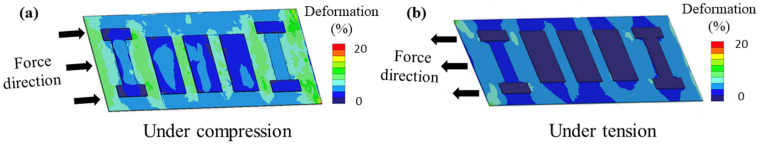
Finite element analysis of flexible biosensor. (**a**) Compression state. (**b**) Tensile state.

Here, *P* is the pressure exerted on the biosensor, ν is the Poisson’s ratio, and *c* is the deflection. When the strain force caused by skin deformation is applied to the biosensor, the proper value of deflection can ensure the biosensor’s structural integrity. Through finite element analysis (FEA), we studied the strain distribution of this structure in compression (Figure 2a) and tension (Figure 2b) states, respectively, to prove its highly deformable characteristic.

### 2.3. Signal Acquisition and Classification

Due to the self-adhesive property and flexibility of the PDMS substrate, the hydrogel biosensor can maintain conformal contact with the dimpled epidermis. In order to collect the horizontal EOG signal and the strain signal generated from the epidermis deformation, the sensor is affixed to the middle of the user’s forehead.

When the eyes move to the left, the right eyeball approaches the inner canthus and transmits a positive potential (500–600 μV) to it, which shows an upward potential spike in EOG waveform [48]. The eye movement to the right transmits a negative potential, and a downward potential spike will appear in EOG waveform. When the eyes move upward or downward, the epidermis of the forehead is compressed or stretched accordingly. In the stretched state, the strain sensor is elongated and its resistance increases; when being compressed, the resistance decreases. The employment of the strain sensor is to complement EOG measurements. Consequently, eye movements can be identified by analyzing the change of potential difference and resistance [49].

**Figure 3:**
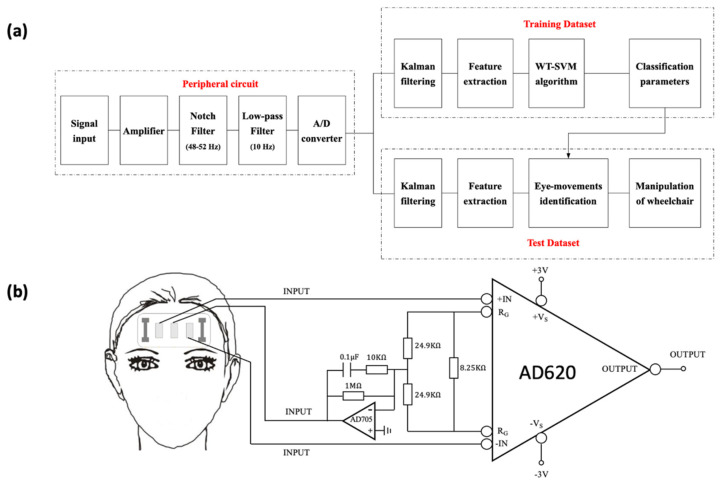
Processing of EOG and strain signals. (**a**) Flow chart of signal acquisition and classification. (**b**) Instrumentation amplifier.

The flow chart of signal acquisition and classification is shown in Figure 3a. Considering that the electrophysiological signal is weak and contains much interference [50], we designed a circuit composed of an instrumentation amplifier (Figure 3b), a notch filter, a low-pass filter, and an A/D conversion module to process it. The instrumentation amplifier is utilized to amplify the signal without reducing the SNR (signal-to-noise ratio) [51]. Since the frequency of power supply is 50 Hz, the notch filter (48–52 Hz stop band) is applied to remove the power frequency interference. The main frequency of the EOG signal is concentrated below 10 Hz, so a low-pass filter is set to filter out the high frequency noise. Before being input into the laptop, the analog signal will be converted into a digital signal through an A/D conversion module.

To further improve the SNR of the signal data, a Kalman filtering algorithm is applied, and the principle of it is illustrated below. The predicted value of the signal (Equation (3)) is determined by the true value of the previous time point. The covariance matrix (Equation (4)) represents the difference between the predicted value and the real value. In order to reduce the error, it is necessary to obtain the value of the Kalman gain (Equation (5)) and continuously correct it during iteration. After obtaining the error covariance matrix and the Kalman gain, the true value of the signal (Equation (6)) can be calculated. The error covariance matrix (Equation (7)) between the estimated value and the true value will be used in the next iteration to obtain a better effect of signal filtering.

(3) $${\hat{X}}_{k}=A{\hat{X}}_{k-1}+BU_{k-1}$$

(4) $$\sum K=A\sum \left(K-1\right)A^{T}+Q$$

(5) $$K^{\prime }=\sum KC^{T}{\left(C\sum KC^{T}+R\right)}^{-1}$$

(6) $${\hat{X}}_{k}^{\prime }=X_{k}+k^{\prime }\left(Z_{k}-C{\hat{X}}_{k}\right)$$

(7) $$\sum K^{\prime }=\sum K-k^{\prime }C\sum K$$

After implementing the Kalman filtering algorithm, the WT-SVM algorithm is introduced to achieve signal classification based on the signal features (amplitude, duration, interval). The filtered signal can be regarded as a superposition of the strain signal and the EOG signal. Due to the distinct characteristics of the two kinds of signals, setting a coefficient threshold of wavelet decomposition can separate them [52,53]. In the process of wavelet decomposition, signals are decomposed into low-frequency and high-frequency parts, and in the next decomposition, the low-frequency part is decomposed into a higher-order low-frequency part and a high-frequency part [54]. The N-order low-frequency signal and the (N + 1)-order low-frequency signal are combined to form the wavelet basis function, and the signals are then reconstructed.

The reconstructed signal data are divided into two data sets: training data set and test data set. There are five typical eye movement states (up, down, left, right, and straight), corresponding to five types of signals. Since the SVM is a two-class classifier, we adopt the “one-against-one” strategy and design ten classifiers to train the signal data. Considering the linear inseparability between the signals, the SVM classifiers use the Gaussian kernel function (Equation (8)) to map the five types of signals to a high-dimensional space to distinguish them.

The characteristic value of a signal is $f\left(\zeta_{i},{{\;\zeta }_{i}}^{\prime }\right)$, and its corresponding weight value is $\phi_{i}$. The SVM classifier can find the appropriate value of $\phi_{i}$ and limit the weighted values of different signals’ eigenvalues (Equation (9)) in a specific interval, and the above four kinds of signals (up, down, left, and right) can then be distinguished. If the weighted value falls on the boundary of the interval exactly, this means that the eye gaze direction is just straight.

(8) $$\mathrm{Gaussian}\;\mathrm{kernel}\;\mathrm{function}\;f\left(x,x^{\prime }\right)=\exp(-\frac{∥x-x^{\prime }{∥}^{2}}{2\sigma^{2}}),\;\;\mathrm{where}\;x=\zeta_{i}$$

(9) $$V=\overset{n}{\underset{i=1}{\sum }}f\left(\zeta_{i},{\zeta_{i}}^{\prime }\right)\phi_{i}\{\begin{array}{c} upward,\;V\in \left(0,1\right)\\ downward,\;V\in \left(-1,0\right)\\ left,V\in \left(1,2\right)\;\\ right,\;V\in \left(-2,-1\right) \end{array}$$

## 3. Experimental Results Analysis

### 3.1. Performance of Flexible Hydrogel Biosensor

**Figure 4:**
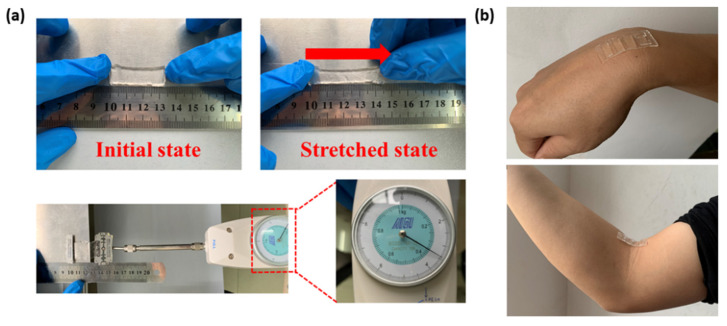
Performance test of the flexible hydrogel biosensor. (**a**) Tensile test. (**b**) Biosensor–epidermis adhesion.

We have conducted a series of experiments to study the performance of the biosensor to verify the feasibility of its application in continuous electrophysiological signal monitoring. The mechanical performance of the biosensor is reflected in its structural flexibility, so we studied the relationship between the tensile force exerted on biosensor and its deformation degree. During the experiment, we used metal clips to clamp the two ends of the membrane. One end was fixed and aligned to a certain scale of a steel ruler, and the other end was connected to a tension gauge (NK-10, Hongkong Aigu Instrument Co., Ltd., Hongkong, China) (Figure 4a). The exceptional mechanical property of the biosensor enables it to follow the deformation of the epidermis without structural damage (Figure 4b).

In further experiments, the measurement of Young’s modulus was realized on a stretcher (DHY-2, Minsks Testing Equipment Co., Ltd., Xi'an, China). The biosensor was clamped to the platform of the stretcher and tensile force was exerted on it through clips. After reading the value of force, the Young’s modulus of biosensor was obtained according to Equation (10).

As to the measurement of the electrical behavior of biosensor, the method of voltammetry is introduced to determine the resistance and the conductivity [50]. We fixed both ends of the biosensor and applied a voltage to it through a power supply (UTP1306S, Unitech Technology Co., Ltd., Dongguan, China). Then, we stretched the biosensor to different lengths and recorded the corresponding current and voltage values. By measuring the width and thickness of the biosensor, the value of conductivity could be calculated by Equation (11).

(10) E=F∗L/(A∗∆L)

(11) $$\rho =U_{t}*S/\left(I_{t}*L\right)$$

To ensure the comfortable wearing of the biosensor, we have carried out research into its breathability. Three glass bottles were filled with same amount of water: one opened naturally, one was covered with a biosensor at the mouth of the bottle, and the other was covered with a traditional dry electrode. The evaporation of water through the biosensor resembles the evaporation process of sweat. After several hours, the breathability of biosensor was evaluated by calculating the water loss.

**Figure 5:**
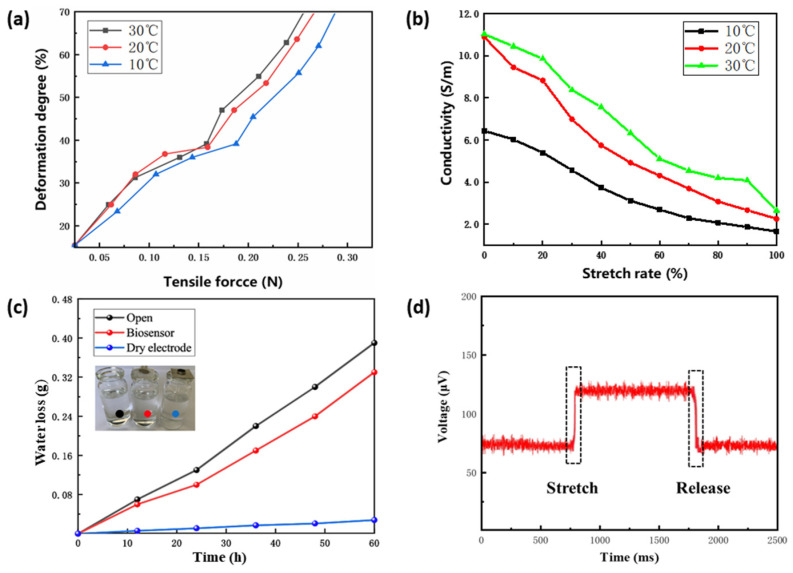
Measurement of characterization. (**a**) Mechanical behavior. (**b**) Electrical behavior. (**c**) Comparison of breathability. (**d**) Response time.

The relationship between the tensile force and the degree of deformation is presented in Figure 5a. The deformation of the biosensor changes slowly as tensile force increases at the beginning. When the tensile force increases to a certain value, the biosensor deforms significantly due to the change of internal structure. Furthermore, at a lower temperature, deformation caused by the same tensile force is smaller. In the environment of 30 °C, under the tensile force of 0.07 N, the deformation degree was approximately 20%, which is close to the maximum deformation of human skin without tearing [55]. We observed the surface of the biosensor with a microscope (LIOO, S600T) and found no obvious structural fractures. Experimental measurements found that the Young’s modulus of the biosensor is 286 kPa, which can fully adapt to the deformation of skin.

As to the electrical properties, Figure 5b displays the conductivity of the biosensor at different stretch rates. The elongation of the biosensor leads to the increment of its resistance and the reduction of its conductivity. When the stretch rate is in the range of 0–20%, the conductivity decreases more remarkably. High temperature intensifies the movement of ions inside the hydrogel membrane, which explains why, in the environment of 20 °C and 30 °C, the conductivity is significantly greater than that in 10 °C.

The conformal contact between the biosensor and the epidermis leads to a reduction in contact resistance. We measured the skin contact resistance of the proposed biosensor and the Ag/AgCl electrode separately (26 °C, 60% humidity). Two electrodes were attached to the forehead epidermis, and after connecting to the same AC signal source, they are led out to the input of an instrumentation amplifier. The amplitude of the AC signal was 100 μA and the frequency was fixed at 100 Hz. Thus, the skin contact resistance can be obtained according to the voltage difference. The skin contact resistance of the flexible biosensor is 39.6 KΩ, which is smaller than that of the Ag/AgCl electrode (43.2 KΩ).

In the breathability experiment, the flexible biosensor showed a water vapor transmission rate (WVTR) of 18 g m^−2^ h^−1^ (Figure 5c), which is close to that of epidermis (20 g m^−2^ h^−1^). This indicates that the biosensor has great breathability and will not cause discomfort when laminating on the epidermis. Additionally, the biosensor delivers a high sensitivity of 7.8 mV·N^−1^ and a fast response time of 103 ms (Figure 5d).

The ductility of the sensor is the key to maintaining conformal contact with the skin during long-term monitoring. We compared the performance of the flexible biosensor and sensors that made from other materials in Table 1, which illustrates that why we choose HPC/PVA to fabricate the flexible biosensor.

### 3.2. Eye Movements Identification

**Figure 6:**
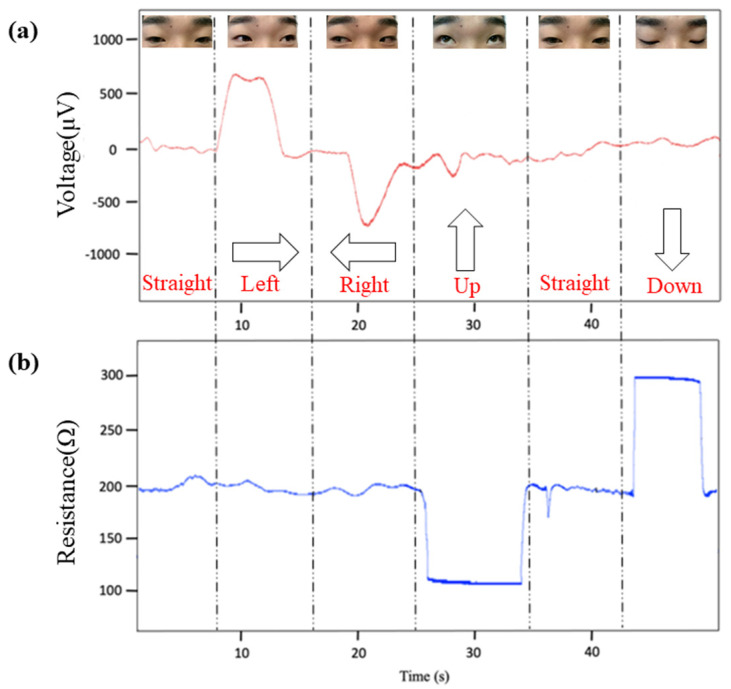
Presentation of the electrophysiological signals in diverse eye movement states. (**a**) EOG signal. (**b**) Strain signal.

The waveforms of EOG and strain signals corresponding to various eye movements are depicted in Figure 6. To determine the recognition accuracy of the WT-SVM algorithm for eye movements, we collected 600 sets of eye movement signals from 30 volunteers with normal vision.

We recorded the actual states and the states identified by algorithms and constructed a 5×5 determinant. The value of each term in the determinant is the count that a particular state is recognized as another state (for example, the value of first row and first column of the determinant ($a_{11}$) is the count that the actual straight state is recognized as the right state). The correct recognition rate of each state (straight, upward, downward, left, and right) and the comprehensive accuracy can be calculated by Equations (12) and (13), respectively.

(12) $$S_{n}=a_{\left(6-n\right)n}/\sum_{i=1}^{5}a_{in}\;\;\;\;\;\;\;\left(n=1,2\ldots 5\right)$$

(13) $$accuracy=0.2*\sum_{n=1}^{5}S_{n}$$

**Figure 7:**
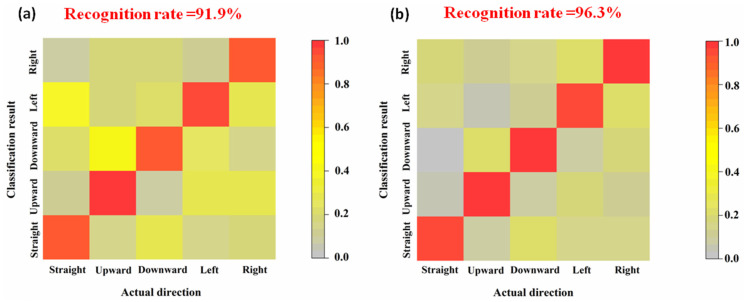
Recognition accuracy of eye movement states. (**a**) Ag/AgCl electrode. (**b**) Flexible hydrogel biosensor.

The classification and recognition result of signal data collected by Ag/AgCl electrode and flexible hydrogel biosensor are presented in Figure 7.

**Figure 8:**
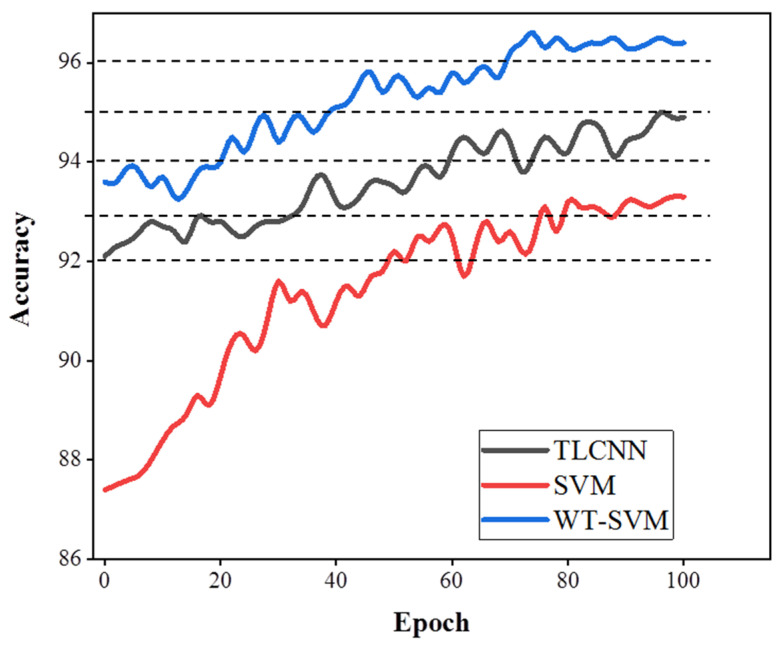
Accuracy comparison of classification algorithms (TLCNN, SVM, WT-SVM).

As can be seen in Figure 8, in the first epoch, the accuracy of the WT-SVM algorithm reached 93.6%. Compared with TLCNN and the traditional SVM method, it is fully trained and achieves a stable accuracy in less epochs. Therefore, the results of the WT-SVM algorithm in eye movement recognition are reproducible.

### 3.3. In-Site Experiment

**Figure 9:**
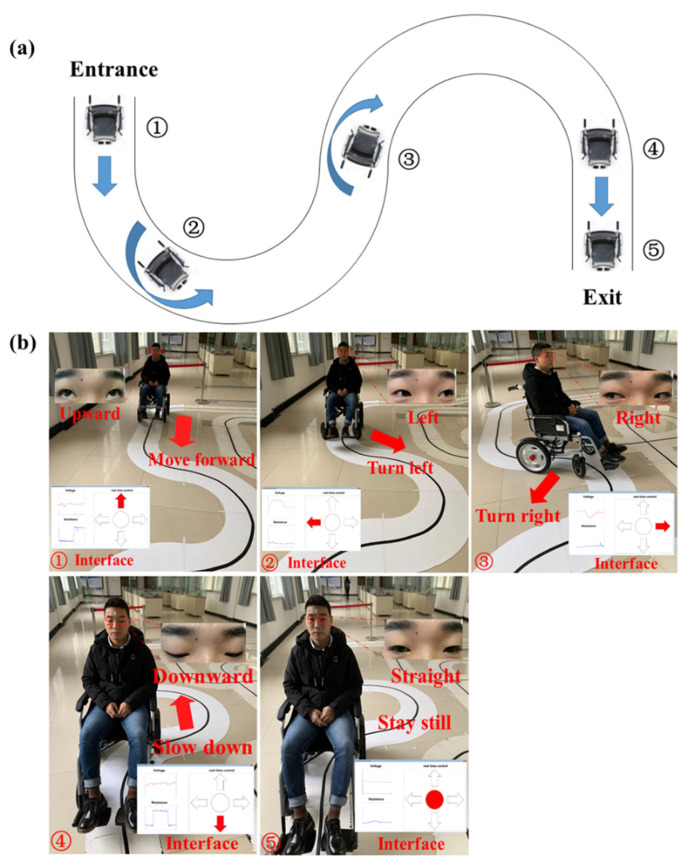
In-site experiment of eye movement-controlled wheelchair prototype. (**a**) Route map of the bend. (**b**) Diverse eye movement states and corresponding wheelchair movement modes.

We built a bend (Figure 9a) on an open field to examine the practicability of this eye-movement-controlled wheelchair prototype. Ten volunteers participated in this test and they were told how to control the wheelchair through eye movements. After an average of half an hour, they could already manipulate the wheelchair to their wills.

To pass the bend without collision, firstly, the selected volunteer needs to look upward to drive the wheelchair forward. When reaching the turning point, gazing at left or right will control the turning of wheelchair. Lastly, the tester should look downward at the exit to slow down the wheelchair gradually until it stops (Figure 9b). Ultimately, nine volunteers successfully maneuvered the wheelchair through the bend, which shows a certain degree of usability of the bioelectric system.

## 4. Conclusions

This work provides a new idea for realizing the eye-movement-control of wheelchairs through the combined analyses of EOG and strain signals. Owing to its structural flexibility, the hydrogel biosensor can be well-adhered to the dimpled epidermis, which allows it to collect electrophysiological signals accurately. Compared to the rigid Ag/AgCl electrode, the flexible biosensor is more biocompatible and less likely to cause skin irritations. To process and classify the signal data, Kalman filtering and WT-SVM algorithms are introduced. This soft bioelectronic system demonstrates a 96.3% accuracy for eye movement recognition, which is higher than the traditional rigid system (91.9%). The proposed eye movement control method possesses broad application scenarios like industrial control and AR/VR field.

## Figures and Tables

**Table 1:** A summary of sensor’s characterization. CNT—carbon nanotube; PEDOT:PSS—poly(3,4-ethylenedioxythiophene):poly(styrenesulfonate).

Materials	Stretchable Sensitivity	Young’s Modulus	Ref.
CNTs/PDMS	5.1 mV·N^−1^	445 KPa	[56]
PEDOT:PSS	0.4 μV·N^−1^	2 × 10^6^ KPa	[57]
Graphene	83.9 μV·N^−1^	9 × 10^8^ KPa	[58]
HPC/PVA	7.8 mV·N^−1^	286 KPa	Our work

## Data Availability

Not applicable.
